# Using implementation science to mitigate worsening health inequities in the United States during the COVID-19 pandemic

**DOI:** 10.1186/s12939-020-01293-2

**Published:** 2020-10-01

**Authors:** Tyler A. Jacobson, Lauren E. Smith, Lisa R. Hirschhorn, Mark D. Huffman

**Affiliations:** 1 Department of Preventive Medicine, Northwestern University Feinberg, School of Medicine, 710 N. Lake Shore Drive, Suite 800, Chicago, IL 60660 USA; 2 Department of Medical Social Sciences, Northwestern University Feinberg School of Medicine, Chicago, IL USA; 3 The George Institute for Global Health, Sydney, Australia

**Keywords:** Covid-19, Social distancing policies, Implementation outcomes, Implementation science, Health inequities, Testing, Contact tracing

## Abstract

With the threat of coronavirus disease 2019 (Covid-19) enduring in the United States, effectively and equitably implementing testing, tracing, and self-isolation as key prevention and detection strategies remain critical to safely re-opening communities. As testing and tracing capacities increase, frameworks are needed to inform design and delivery to ensure their effective implementation and equitable distribution, and to strengthen community engagement in slowing and eventually stopping Covid-19 transmission. In this commentary, we highlight opportunities for integrating implementation research into planned and employed strategies in the United States to accelerate reach and effectiveness of interventions to more safely relax social distancing policies and open economies, schools, and other institutions. Implementation strategies, such as adapting evidence-based interventions based on contextual factors, promoting community engagement, and providing data audit and feedback on implementation outcomes, can support the translation of policies on testing, tracing, social distancing, and public mask use into reality. These data can demonstrate how interventions are put into practice and where adaptation in policy or practice is needed to respond to the needs of specific communities and socially vulnerable populations. Incorporating implementation research into Covid-19 policy design and translation into practice is urgently needed to mitigate the worsening health inequities in the pandemic toll and response. Applying rigorous implementation research frameworks and evaluation systems to the implementation of evidence-based interventions which are adapted to contextual factors can promote effective and equitable pandemic response and accelerate learning both among local stakeholders as well as between states to further inform their varied experiences and responses to the pandemic.

## Introduction

After widespread and varied implementation relaxation of shelter-in-place policies across the United States (U.S.), questions of when and how best to relax or (re)tighten social distancing policies remain relevant. Socioeconomic and political pressures have been mounting to “open the economy.” However, without clear evidence-based plans to evaluate and expand prevention, detection, isolation, and treatment, states and counties risk not only lifting restrictions prematurely but also reinstating them late, leading to a resurgence of coronavirus disease 2019 (Covid-19) cases on top of already-stressed health systems.

Social distancing policies in the U.S. are being relaxed in phases, and state- and county-level case counts, regional hospital and intensive care unit capacity, testing, and contact tracing are recommended metrics to inform these policies and their timing [1]. Implementation science methods have been developed to design, evaluate, and adapt implementation strategies for evidence-based interventions (EBIs) and can be used for varying contexts including different stages of the pandemic [2, 3]. As the Covid-19 pandemic has worsened pre-existing health inequities [4], implementation research is also urgently needed to ensure the implementation of EBIs is both effective and equitable [5].

Implementation research can play a critical role in assisting policymakers and state- and county-level public health departments in effectively designing and implementing policies for pandemic response in their own settings. Implementation research can help identify contextual factors, which require different implementation strategies or adaptations, particularly for historically underserved populations. Measuring implementation outcomes, such as reach, feasibility, acceptability, adoption, fidelity, and maintenance, can help implementers recognize and respond to inequities in the direct impacts of Covid-19 and the effects of policy responses. Lessons learned from implementation successes and failures can allow states and counties to accelerate needed adaptations as compared to using lagging indicators, such as Covid-related hospitalization and mortality rates.

In this commentary, we highlight opportunities to incorporate implementation science into the design and evaluation of interventions needed to more safely relax social distancing policies in the U.S. This commentary specifically explores Covid-19 responses related to: 1) testing and surveillance, 2) contact and location tracing, 3) public mask use, and 4) social distancing, as well as unintended consequences of Covid-19 policies to ensure not only an equitable pandemic response but also a more equitable society in the post-pandemic era.

## Testing and surveillance

### Implementation strategies for effective and equitable testing

Widespread testing is critical to safely re-open communities; however, individual, community, and system-level barriers pose threats to successful implementation [1, 6]. Incorporating implementation outcomes, such as acceptability, feasibility, adoption, and reach, into monitoring and evaluation is needed to drive equitable testing, prioritize communities and workers at greatest risk, and modify testing strategies to state, county, and local contexts (Table 1). Collecting and reporting disaggregated data are needed to inform the need for adapting testing strategies to promote equity in testing among under-represented communities and groups. For example, disaggregated data on the number and locations of testing sites, daily testing rates by site, and sociodemographic characteristics of people tested can be used to evaluate regional or local implementation, reach, and acceptability of testing strategies. Because most variation in overall health status and outcomes is driven by socioeconomic factors linked to demography and geography [7], exploring reasons for variability in Covid-19 testing across key subgroups can provide insights into the contextual barriers and facilitators to testing, which can inform adaptations to improve these outcomes and reduce transmission overall.

**Table 1:** Using an implementation outcomes framework described by Proctor et al. (2011) [3] to evaluate testing strategies in response to the Covid-19 pandemic

Implementation outcomes	Definitions^**3**^	Evaluation of implementation strategies
**Acceptability**	Perception that the intervention is agreeable, palatable, or satisfactory	• Community attitudes towards testing
		• Documented barriers and facilitators to testing from qualitative surveys and focus groups
	Considered from the perspective of individuals receiving testing	• Input and feedback from community leaders and organizations (not involved in operations) about testing strategies
**Adoption**	The intention to employ or adhere to an intervention	• Number of testing sites and daily testing rate by site
	Considered from the perspective of entities providing testing	• Testing strategies stratified by geographic location and entity
**Appropriateness**	Perceived fit, relevance, or compatibility of intervention for the given setting and problem	• Community and other stakeholders’ attitudes towards local testing strategies
		• Document changes in testing strategies compared to original protocols
**Feasibility**	The extent to which an intervention can be successfully used or carried out in a particular setting	• Input and feedback from entities operating testing
		• Availability of tests given demand (supply chain)
**Fidelity**	The degree to which an intervention was implemented as it was originally intended	• Number of improperly collected, transported, or handled samples
		• Whether test results are returned promptly and confidentially
**Cost**	Cost impact of an implementation effort	• Overall and per-test costs across geographic areas and/or by organizations providing testing
**Penetration (Reach)**	Integration of intervention within a setting or its subsystems	• Number of tests, by test type, across key subgroups^a^ and representativeness of testing†
		• Proportions of communities tested and percent-positive rates in a given time period
**Sustainability (Maintenance)**	How well an intervention is maintained over time within an organization or setting	• Maintenance of testing capacity and performance for population health over time and across key subgroups^ab^

^a^Key subgroups: age, sex, race/ethnicity, language, geographic unit

^b^Explore reasons for variability across key subgroups

To increase acceptability and feasibility of testing, trusted community leaders and community-based organizations must be involved in developing and coordinating testing strategies, including diversity of testing locations [1, 6]. This strategy of stakeholder engagement, an important tool within implementation research, will increase understanding of contextual factors to drive implementation strategy choice and design with regard to access to testing. For example, home-testing diagnostic kits and employer-based screening and testing may not be accessible to all, but can primary care offices, pharmacies, community centers, and community-based outreach be engaged and incentivized to complement these approaches in hard-to-reach (or hardly reached [6]) communities? Drive-thru testing has been widely hailed as an efficient strategy, but can these approaches be adapted for walk-thru (or walk-in) testing for the millions of Americans without cars? Other testing strategies have included sending health workers to households lacking means of transportation and setting up temporary mobile testing sites.

To address the acceptability and appropriateness of testing and surveillance strategies, fostering engagement and public trust in community, city, and state-level response systems and holding them accountable will be necessary. Public-facing dashboards on testing capacity, case numbers, and percent-positive rates, stratified across major sociodemographic groups such as age, sex, race/ethnicity, and geography, can help elected officials and public health departments allocate limited testing capacity and identify “cold spots” where more testing is needed to promote equitable access. However, providing public-facing dashboards will not necessarily guarantee their use. Therefore, implementation strategies will need to be identified and evaluated to ensure ongoing stakeholder engagement and facilitate data usage to drive adaptation of policies and implementation.

## Contact and location tracing

### Manual contact tracing

Contact tracing, widely recommended as a key strategy to contain Covid-19, has been deployed both in domestic (e.g., Massachusetts) and international (e.g., South Korea, Ethiopia, Liberia) settings. The Rockefeller Foundation has urged states to form a large-scale COVID Community Healthcare Corps, which would mobilize hundreds of thousands of health workers and other community members to perform testing, contact and location tracing, sanitation of public spaces, and enforcement of social distancing guidelines [1]. This plan may provide some economic respite to displaced workers and offer a more equitable and acceptable approach to contact tracing [1]. Additional benefits can be realized if the strategy includes screening for medical problems, mental health and social challenges, and food insecurity while addressing urgent needs through linkage to care, social services, and financial support. Implementation science can provide valuable information regarding the relative adoption, acceptability, fidelity, and maintenance of these approaches by eliciting and responding to feedback from contact tracers and those whom they assist.

### Digital tools

Digital tracing technologies using smartphones, similar to what has been developed by Google and Apple [1], have been useful in some East Asian countries for containing Covid-19. While this approach may be lower cost and more easily scalable than manual contact tracing, the acceptability of this approach in the U.S. is uncertain due to cultural and legal environments focused on data privacy [2]. Initial uptake in the U.S. has not been robust but may increase over time. Feasibility, acceptability, and effectiveness should be integrated into the evaluation of implementation strategies to increase uptake among the general population and shield those who may not have access to or may be distrustful towards these technologies. Access to testing will also need to be integrated into contact tracing strategies, since asymptomatic cases will not know to alert the system without more widespread testing.

## Public mask use

As communities re-open, high public adherence to mask usage may be effective in slowing transmission [8]. Concerns over SARS-CoV-2 aerosolization suggest public mask usage, combined with modifying behaviors (e.g., avoiding crowds and enclosed spaces) and increasing ventilation within indoor spaces, may further reduce transmission [8]. Public recommendations regarding mask usage have changed dramatically over the course of the pandemic, and policies regarding their use have been heterogeneously adopted and implemented by states and their citizens, in part due to a significant shift required in cultural norms.

Implementation science can help select strategies that increase the acceptability, feasibility, and adoption of public mask use policies [8]. Future research is needed to investigate how compliance and enforcement of statewide mandates on public mask use differ based on contextual factors. Implementation outcomes from China suggest wearing face masks in public is acceptable, feasible, and reassuring and that barriers may include discomfort, difficulties communicating, and high resource use [8]. More data are needed regarding contextual factors affecting implementation within the U.S. For example, individuals may view mandated mask-use as a threat to their civil liberties. Additional social barriers have included lack of access to masks, as well as harassment and racial profiling of some racial/ethnic groups.

Mandating universal public mask use and running public service announcements can potentially mitigate social harms related to public mask use and may increase acceptability. Public service announcements can also instruct the public on proper mask-wearing to promote fidelity. States, counties, and local municipalities can address equity considerations by providing face masks to groups with limited resources [8]. Disparities in local capacities to increase public mask use could exacerbate inequities by providing varying levels of risk for both workers and customers as social distancing policies are relaxed. Monitoring implementation outcomes regarding public mask use will be critical in adjusting policies and implementation strategies as needed.

## Social distancing policies

### Implementation challenges related to self-isolation

While self-isolation remains a key tenant of Covid-19 containment efforts in the U.S., acceptability and feasibility of such recommendations must be addressed to increase adherence and reduce transmission. In China, cases identified by widespread testing were isolated in quarantine facilities (large-scale temporary hospitals known as fangcang isolation shelters) [9]. In the U.S. and Europe, policies have centered around voluntary home self-isolation, in part due to a lack of social acceptability around isolation shelters [9]. Self-isolation poses challenges for people experiencing homelessness who are unable to physically distance in crowded congregate shelters. Lessons learned from abroad can be brought back to the U.S., such as using existing infrastructure for self-isolation and integrating these services into the overall health system for basic medical care and rapid referrals [9]. Cities, states, and municipalities may consider providing voluntary hotel or dormitory stays for individuals experiencing homelessness or who are otherwise unable to self-isolate or quarantine at home, as well as for individuals with household members at high-risk for poor outcomes [10]. Providing income support may also increase acceptability of self-isolation; a study in Israel found that income support increased compliance to self-quarantine recommendations from 57 to 94% [11].

In addition, implementation strategies for self-isolation may need to include legislation to prevent people from losing employment or risking deportation based on infection status [1]. Guaranteeing paid sick leave for those who test positive may also be needed, since lost wages could deter low-income families from seeking testing and care. In communities with a high prevalence of undocumented workers, emphasizing privacy and finding alternative ways to support sick leave will be important [1, 11]. Implementation strategies, such as leveraging manual contact tracers from the community, including street medicine providers [10], to connect individuals with these resources, could increase community perceptions around self-isolation options, educate individuals on how to properly self-isolate, and inform them how to monitor and seek care when necessary. Responding to implementation outcomes, such as acceptability and feasibility, for strategies that increase adherence to self-isolation will assist policymakers and public health institutions in recognizing barriers, adapting implementation as needed, and reducing both community and within-household transmission.

### Human-centered design for social distancing

Creative design solutions and systems engineering are needed to reduce the cognitive load for individuals and ease the adoption of social distancing practices in public spaces. Intentional re-design of public spaces can clearly instruct individuals on how to follow social distancing guidelines. These solutions have already been adopted, such as in stores only allowing customers to walk down aisles in one direction, or in parks that mark 6-ft distances for individuals and small groups to reduce their risk of exposure. Local public health officials will need to partner with community leaders and business owners to monitor data regarding the acceptability and fidelity of re-design solutions and adapt interventions as needed to promote safety. Trusted community leaders must lead efforts to identify where such design solutions are needed and assist in development. Special considerations should be given to ensure design solutions are implemented equitably in urban and rural environments. Modifications may also be needed to ensure designs are easily understandable regardless of language or literacy.

### Strategies to mitigate transmission in under-resourced communities

As social distancing policies are relaxed, mitigation strategies to support under-resourced communities are needed to prevent outbreaks and wider second surges. For example, how might temperature or infrared screenings be promoted in small businesses, schools, houses of worship, and community centers, particularly in hard-hit or under-resourced communities? Will sufficient personal protective equipment be available and at what cost to protect workers, visitors, and community members? Other mitigation strategies, such as decreasing office, retail, and restaurant capacities to increase safety, may not be sustainable for economically disadvantaged businesses. Recognizing both barriers and facilitators to implementation will be critical towards selecting and adapting precautionary measures to local contexts and capacities, especially in under-resourced communities.

### Re-tightening social distancing policies

Spikes in Covid-19 cases have been predicted in late fall 2020 and winter 2020–2021, which may require re-tightening social distancing policies in the absence of successful EBI implementation. Transparency in data not only related to Covid-19 cases but also to implementation outcomes may increase acceptability of re-tightening social distancing policies, especially if triggers to return to more stringent social distancing are explicitly defined and communicated. If policies revert back to stricter phases, high public adherence and implementation of EBIs will depend upon the public perceiving these policies as appropriate to address the Covid-19 pandemic overall and in their communities. Implementation strategies may include efforts to educate the public about the benefits of these policies through a combination of mass media, social media, and communication briefs disseminated by community-based organizations and other key opinion leaders.

## Unintended consequences of COVID-19 policies

As a result of shelter-in-place orders, increases in domestic violence, alcohol consumption, food insecurity, and exacerbation of mental health issues, including increased suicide risk, have been reported [1, 12, 13]. Depression, anxiety, and post-traumatic stress can develop in people who have developed Covid-19 or who have lost family members to the pandemic [12]. These burdens are disproportionately affecting Black and Latinx communities, which are contracting SARS-CoV-2 more frequently and dying at higher rates [4]. Flexible and tailored mental health services and assessments as well as strong community support are needed [12].

Delays in access to other EBIs critical to reducing morbidity and mortality beyond Covid-19, including vaccinations, early diagnosis, and care for chronic conditions, may lead to the presentation of more severe illness and exacerbate health inequities both during and after the pandemic. School closures also disrupt the lives of children and are likely to affect child well-being. These measures may also heighten food insecurity, particularly among lower-income families [13]. Children living in poverty or who are homeless can face significant difficulties with e-learning, and those who depend on school-provided meals can face significant food insecurity. Consequently, school closures and mounting economic adversity are likely to widen the academic achievement gap [13].

Implementation research can help to better understand the causal link between Covid-19 policies and these unintended consequences by measuring and exploring proximate factors. Incorporating these indirect effects into implementation research is critical to selecting and designing equitable implementation strategies. For example, epidemiological and simulation modeling studies can identify optimal implementation strategies or adaptations that minimize the predicted toll of the pandemic on disadvantaged communities, while also mitigating indirect effects which are likely to worsen health disparities [5].

Continuously adapting EBI implementation may be necessary to mitigate these indirect effects. The accumulation of indirect effects could make implementation more difficult over time by lowering the acceptability and adoption of such policies. The indirect effects of social distancing policies are also likely to be long-lasting, which could adversely affect later stage implementation outcomes, such as penetration and sustainability. Consequently, strategies to mitigate the indirect effects are also strategies to improve policy design and implementation.

## Conclusion

Determining when and how to begin relaxing social distancing policies in the United States has been challenging and should be informed by implementation research methods to mitigate worsening health inequities and accomplish an effective containment strategy. Implementation strategies must be intentionally designed to achieve health equity in testing and surveillance, contact and location tracing, public mask use, and social distancing. With broad and inclusive dissemination of effective implementation strategies for disadvantaged populations [5], policymakers and state and county public health departments can adapt and adopt implementation strategies according to their local contexts, capacity, and time-course in the pandemic. Although successful surveillance for case detection and response will mean the direct consequences of the pandemic may subside, social distancing policies will need to remain in place, perhaps until herd immunity is reached via mass vaccination programs. The indirect effects of social distancing policies, however, are likely to be long-lasting, with disadvantaged populations bearing a disproportionate burden of the unintended consequences from these policies. Efforts to mitigate these consequences must be incorporated into implementation strategies to ensure a more equitable society in the post-pandemic era.

## Data Availability

Not applicable.
